# PICK1 regulates the trafficking of ASIC1a and acidotoxicity in a BAR domain lipid binding-dependent manner

**DOI:** 10.1186/1756-6606-3-39

**Published:** 2010-12-21

**Authors:** Wenying Jin, Chong Shen, Lan Jing, Xiang-ming Zha, Jun Xia

**Affiliations:** 1 Division of Life Science, The Hong Kong University of Science and Technology, Clear Water Bay, Kowloon, Hong Kong, China; 2 Department of Neurobiology, Institute of Neuroscience, Zhejiang University School of Medicine, 388 Yu Hang-tang Road, Hangzhou, Zhejiang 310058, China; 3 Department of Cell Biology and Neuroscience, University of South Alabama College of Medicine, Mobile, AL 36688, USA; 4 State Key Lab of New Drug & Pharmaceutical Process, Shanghai Institute of Pharmaceutical Industry, 1320 West Beijing Rd, Shanghai 200040, China

## Abstract

**Background:**

Acid-sensing ion channel 1a (ASIC1a) is the major ASIC subunit determining acid-activated currents in brain neurons. Recent studies show that ASIC1a play critical roles in acid-induced cell toxicity. While these studies raise the importance of ASIC1a in diseases, mechanisms for ASIC1a trafficking are not well understood. Interestingly, ASIC1a interacts with PICK1 (protein interacting with C-kinase 1), an intracellular protein that regulates trafficking of several membrane proteins. However, whether PICK1 regulates ASIC1a surface expression remains unknown.

**Results:**

Here, we show that PICK1 overexpression increases ASIC1a surface level. A BAR domain mutant of PICK1, which impairs its lipid binding capability, blocks this increase. Lipid binding of PICK1 is also required for PICK1-induced clustering of ASIC1a. Consistent with the effect on ASIC1a surface levels, PICK1 increases ASIC1a-mediated acidotoxicity and this effect requires both the PDZ and BAR domains of PICK1.

**Conclusions:**

Taken together, our results indicate that PICK1 regulates trafficking and function of ASIC1a in a lipid binding-dependent manner.

## Background

Acid-sensing ion channels (ASICs) are a group of cation channels that are activated by a decrease in extracellular pH [1,2]. Four ASIC genes (*ACCN1-4*) have been identified, with ASIC1 and ASIC2 each has two splice variants (a and b). Each ASIC subunit contains two transmembrane domains with both N- and C-termini reside inside the cell and a large cysteine-rich extracellular domain. Recent crystal structure shows that ASICs are trimers [3].

ASICs are predominantly expressed in the nervous system. In the central nervous system, ASIC1a is the major subunit determining acid-activated responses in neurons [4,5]. ASIC1a localizes to dendritic spines and regulates acid-induced Ca^2+ ^increase in spines [6,7]. ASIC1a contributes to synaptic plasticity, learning and fear [5,8-10]. More importantly, ASIC1a plays critical roles in multiple neurological diseases including ischemia [11,12], multiple sclerosis [13], Parkinson's disease [14], seizure [15], and pain [16,17]. These studies demonstrate the importance of ASIC1a in diseases. Several studies have examined the modulation of ASIC1a channel activity by redox reagents, divalent ions, and peptides [18-24]. However, molecular mechanism regulating ASIC1a trafficking is not well understood.

PICK1 (protein interacting with C kinase 1) is a scaffolding protein that regulates trafficking of multiple membrane proteins [25]. For example, interaction of PICK1 with GluR2, an AMPA-type glutamate receptor subunit, is important for synaptic targeting and surface expression of AMPA receptors during synaptic plasticity [26-33]. PICK1 also regulates vesicle trafficking between Golgi and acrosome in spermatids and deficiency of PICK1 in mice leads to abnormal acrosome formation and male infertility [34]. Two domains of PICK1 are important for its function. Its PDZ (PSD-95/Dlg/ZO-1) domain mediates direct interaction with many proteins that contain a PDZ binding motif. In addition to the PDZ domain, the middle portion of PICK1 contains a BAR (Bin/amphiphysin/Rvs) domain [35], which directly binds to lipids, mainly phosphoinositols [36]. Both the PDZ and BAR domains work together and enable PICK1 to couple its PDZ domain-binding partners (e.g. GluR2) to protein trafficking machinery [36,37].

Previous studies show that ASIC1a interacts with PICK1 and this interaction changes the subcellular clustering of ASIC1a [38,39]. The interaction of ASIC1a and PICK1 is mediated by the C-terminus of ASIC1a and the PDZ domain of PICK1. Furthermore, the interaction of PICK1 with ASIC1a was found to be regulated by protein kinases [40,41]. However, whether PICK1 regulates ASIC1a trafficking remains unknown.

Given that PICK1 regulates trafficking of several membrane proteins, and that PICK1 directly interacts with ASIC1, we hypothesize here that PDZ and BAR domains of PICK1 cooperatively regulate ASIC1a surface expression. Our results showed that PICK1 regulates cell-surface expression of ASIC1a in a lipid binding-dependent manner. Further, we showed that this interaction regulates acidosis-induced cell toxicity.

## Results

### PICK1 regulates surface levels of ASIC1a

**Figure 1:**
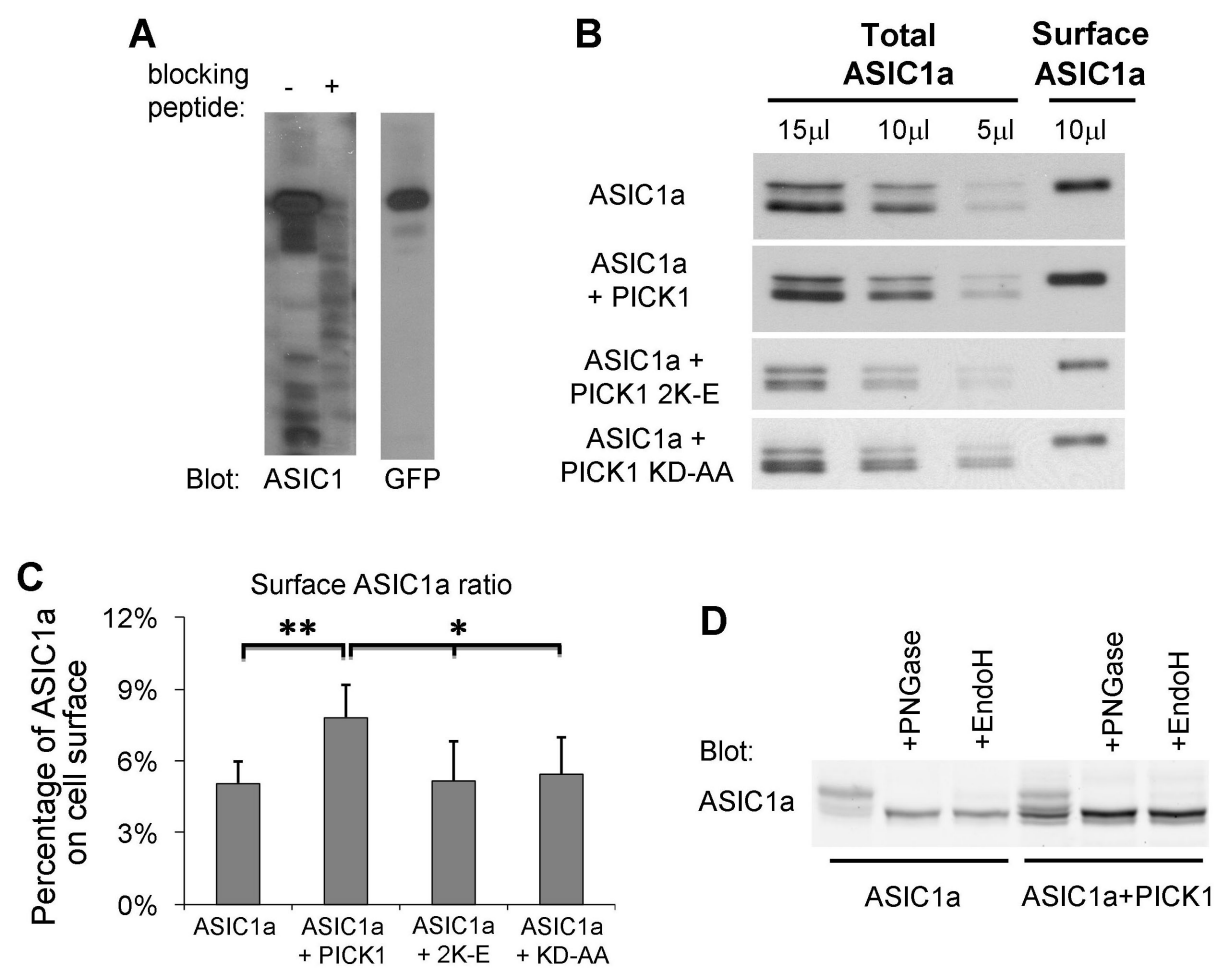
**PICK1 increases surface levels of ASIC1a**. ***A***, HEK293T cells were transfected with GFP-ASIC1a and cell lysates were subjected to Western blot analysis. Purified antibody detected a strong band in cells transfected with ASIC1a and this band was blocked by pre-absorbing with antigen ASIC1-CT (ASIC1a C-terminal 60 amino acids fusion protein). As a position control, GFP antibody detected a band at the same position, indicating this band is GFP-ASIC1a. ***B***, ASIC1a alone or ASIC1a together with either wild-type or mutant PICK1 were transfected into HEK293T cells as indicated. Surface ASIC1a proteins were isolated using surface biotinylation assay. Total and surface samples were resolved by SDS-PAGE and immuno-blotted with the same ASIC1 antibody shown in *A*. Co-expression with PICK1 increased the band intensity of surface ASIC1a. ***C***, Quantification data from multiple experiments in *B*. Surface ASIC1a ratios, expressed as the percentage of ASIC1a on the surface relative to total ASIC1a, were determined by fitting surface ASIC1a to a standard curve obtained by quantifying different amounts of total ASIC1a. Wild-type PICK1 significantly increased surface ASIC1a (** p < 0.01). Both the BAR domain mutant PICK1 2K-E and the PDZ domain mutant PICK1 KD-AA abolished PICK1-induced increase in surface ASIC1a. ***D***, The effect of PICK1 on glycosylation of ASIC1a. Mouse ASIC1a was transfected alone or together with PICK1 into CHO cells and the lysates were treated with PNGase F or EndoH and analyzed by Western blot. PICK1 increased the species of faster migrating ASIC1a on the gel. PNGase F treatment reduced almost all slowly migrating ASIC1a to the faster migrating species. In contrast, there was still a small fraction of slowly migrating species left with EndoH treatment.

As an ion channel, the number of ASIC1a at cell surface will determine the magnitude of ASIC1a-mediated response. To examine if PICK1 regulates the level of ASIC1a at cell surface, we performed cell surface protein biotinylation assay to measure the surface level of human ASIC1a. ASIC1a or ASIC1a with PICK1 were transfected into HEK293T cells. Surface proteins were labeled with membrane-impermeable biotin and then precipitated from cell lysates with immobilized NeutrAvidin beads. Surface and total samples were subjected to Western blot analysis. Figure 1A shows the validation of an ASIC1a antibody, which was directed against the C-terminal 61 amino acids of ASIC1a. As shown in Figure 1B, PICK1 significantly increases surface expression of ASIC1a. To obtain a quantitative measurement of PICK1's effect on surface ASIC1a, we normalized surface ASIC1a to total ASIC1a and obtained the ratio of ASIC1a at cell surface. This was done by loading different amounts of total ASIC1a to obtain a standard curve. The amount of surface ASIC1a was calculated by fitting the intensity of the surface ASIC1a to the standard curve. As shown in Figure 1C, we found that there was 5.05% ± 0.9% of ASIC1a located at cell surface. Co-expressing with PICK1 increased ASIC1a surface/total ratio to 7.80% ± 1.4% (n = 5, p < 0.01, paired *t*-test).

We also noticed that there were multiple species of ASIC1a on the gel. To answer if this effect is specific to human ASIC1a, we studied mouse ASIC1a. Co-expressing of PICK1 with mouse ASIC1a also increased the population of faster migrating ASIC1a (Figure 1D). Previous studies show that differential glycosylation leads to the formation of multiple species of ASIC1a with different apparent molecular weights on western blots [9,42]. To assess if glycosylation results in the difference observed here, we treated ASIC1a transfected lysates with PNGase F, which removes all N-glycan, or EndoH, which removes high-mannose N-glycans that are added early in the secretory pathway [42,43]. PNGase treatment reduced all slower migrating species to the faster migrating species in both control and PICK1 overexpressing conditions. In contrast, there was still a fraction of slow migrating species after EndoH treatment. These data suggest that PICK1 regulates the glycosylation and/or maturation of ASIC1a.

### PICK1-regulated surface expression of ASIC1a is dependent on the lipid binding of PICK1's BAR domain

The BAR domain of PICK1 binds to lipids and this lipid binding regulates surface expression of AMPA receptors [36]. We therefore asked whether the effect of PICK1 on ASIC1a surface expression is also dependent on the BAR domain of PICK1. PICK1 2K-E is a lipid binding-deficient mutant with two critical residues of the BAR domain, Lys266 and Lys268, mutated to glutamate [36]. Unlike the wild-type PICK1, PICK1 2K-E did not increase surface levels of ASIC1a (surface/total ASIC1a ratio: ASIC1a alone: 5.05% ± 0.9%; ASIC1a + PICK1: 7.80% ± 1.4%; ASIC1a + PICK1 2K-E: 5.18% ± 1.6%, n = 5, Figure 1C). This result indicates that lipid binding is required for PICK1-regulated surface expression of ASIC1a.

To test if PICK1's effect on ASIC1a surface expression requires their interaction, we also examined the effect of a PICK1 PDZ domain mutant, KD-AA, which has two critical residues in the PDZ domain of PICK1, Lys27 and Asp28, mutated to alanine and renders the mutated PICK1 unable to interact with ASIC1a [38,39]. As expected, PICK1 KD-AA had no effect on ASIC1a surface levels (surface ASIC1a ratio with PICK1 KA-AA: 5.42% ± 1.6%, p < 0.05 comparing to ASIC1a + PICK1, not significant comparing to ASIC1a alone or ASIC1a + PICK1 2K-E, n = 4, Figure 1C). These results indicate that both the PDZ and BAR domains are required for PICK1 to regulate ASIC1a surface expression.

### Lipid binding is required for PICK1-induced clustering of ASIC1a

**Figure 2:**
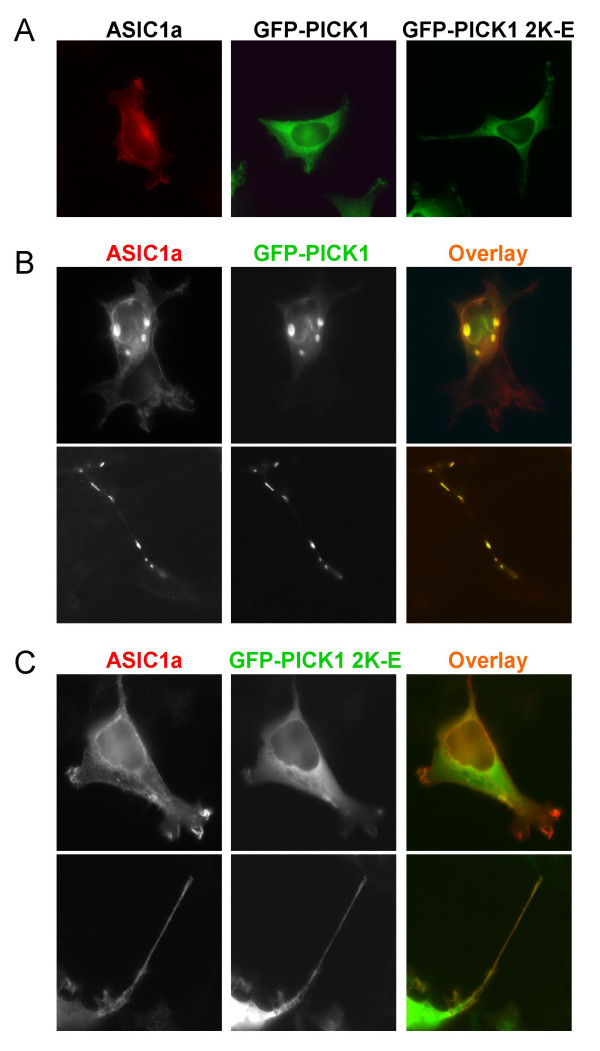
**Lipid binding is required for PICK1-induced clustering of ASIC1a**. ***A***, ASIC1a and wild-type GFP-PICK1 or lipid binding-deficient mutant PICK1 2K-E were transfected into HEK293T cells. The cells were fixed and purified anti-ASIC1 antibody was used to stain ASIC1a. When transfected alone, ASIC1a, PICK1 or mutant PICK1 were all diffusely distributed throughout the cell. ***B***, ASIC1a and GFP-PICK1 were co-transfected into HEK293T cells and stained with anti-ASIC1 antibody. Wild-type PICK1 and ASIC1a formed co-clusters in the cells. While some cells had big co-clusters in the perinuclear site of the cell (upper panel), some cells showed small co-clusters along the cellular protrusions (lower panel). *C*, lipid binding-deficient mutant PICK1 2K-E did not form any co-clusters with ASIC1a in cell body or cellular protrusions.

Next, we asked whether lipid binding of PICK1 affects ASIC1a subcellular localization. We co-transfected ASIC1a with wild-type PICK1 or BAR-domain mutant PICK1 2K-E into HEK293T cells. When expressed alone in 293T cells, ASIC1a was diffusely localized in cytosol with a pattern typical of membrane proteins. Similarly, wild-type PICK1 and PICK1 2K-E were also diffusely localized in cytosol (Figure 2A). When ASIC1a and wild-type PICK1 were co-transfected into 293T cells, they formed co-clusters in the perinuclear region (Figure 2B, upper panel). This localization pattern is similar to what has been reported for PICK1 and ASIC2 [38,39]. One interesting phenomenon we observed was that ASIC1a and PICK1 also formed co-clusters along some cellular protrusions (Figure 2B, lower panel). The nature of these co-clusters is not clear at the moment. In contrast to wild-type PICK1, when PICK 2K-E mutant was co-transfected with ASIC1a in 293T cells, we did not observe any cluster formation (Figure 2C). This result indicates that PICK1 requires its lipid binding ability to regulate the subcellular localization of ASIC1a.

**Figure 3:**
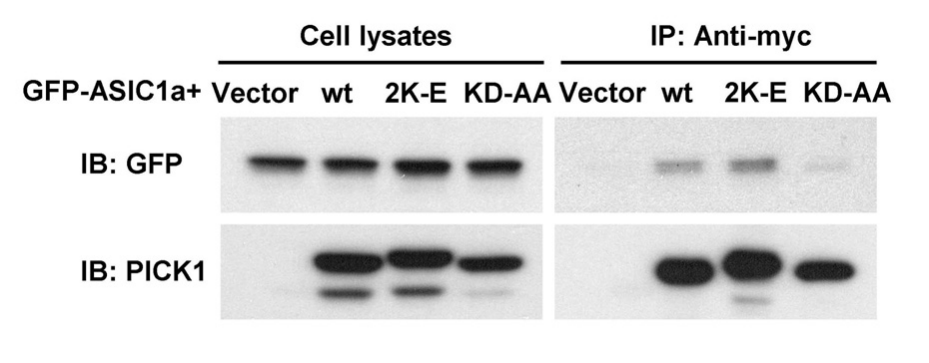
**BAR domain mutant PICK1 2K-E still interacts with ASIC1a**. HEK293T cells were transfected with GFP-ASIC1a together with myc-tagged wild-type PICK1, PICK1 2K-E, PICK1 KD-AA or the vector control, as indicated on the top. Cell lysates were immunoprecipitated with an anti-myc antibody and immunoblotted with either a GFP or a PICK1 antibody as indicated. The BAR domain mutant PICK1 2K-E co-immunoprecipitated with ASIC1a similar to wild-type PICK1 but the PDZ mutant PICK1 KD-AA could not co-immunoprecipitate with ASIC1a.

To answer the question whether mutating the BAR domain of PICK1 interferes with its interaction with ASIC1a, we performed co-immunoprecipitation analysis. GFP-ASIC1a was co-transfected with myc-tagged wild-type or mutant PICK1 into HEK293T cells and immunoprecipitated with an anti-myc antibody. Consistent with earlier studies [38,39], the PDZ domain mutant KD-AA greatly reduced its association with ASIC1a. In contrast, PICK1 2K-E had no effect on ASIC1a:PICK1 interaction (Figure 3). These results suggest that the BAR domain is not required for PICK1 to interact with ASIC1a but is necessary for it to cluster ASIC1a.

### PICK1 regulates ASIC1a-mediated acidotoxicity in a lipid-binding dependent manner

**Figure 4:**
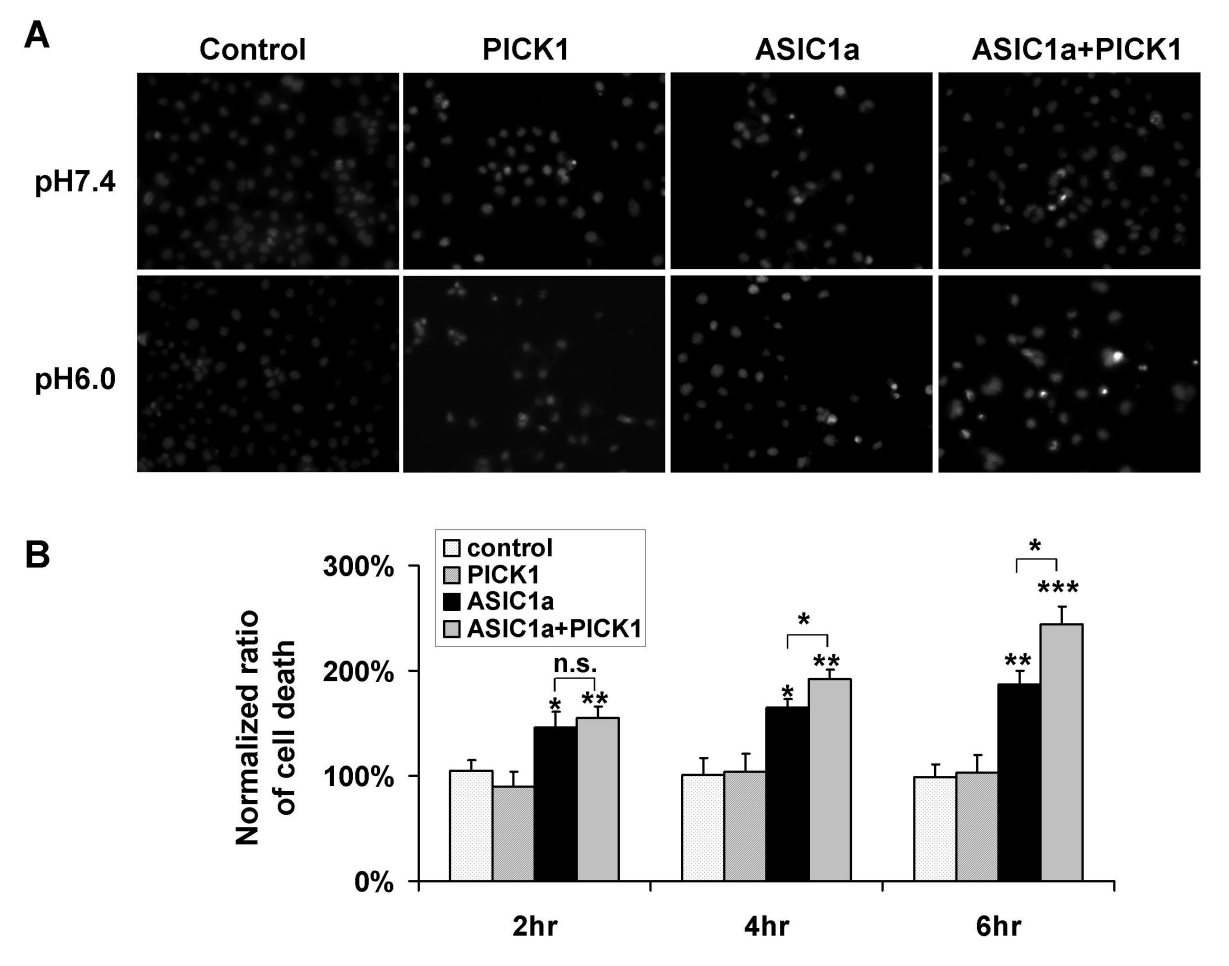
**PICK1 enhances ASIC1a-mediated cell toxicity**. ***A***, Different constructs were transfected into COS7 cells as indicated. The cells were treated with pH7.4 or pH6.0 solution for 2, 4 or 6 hours respectively 48 hours after transfection. Hoechst staining was performed to mark cells undergoing apoptotic cell death, which were identified by their condensed nuclei. The examples after 4 hours of treatment were shown here. There was more cell death in ASIC1a transfected cells compared with non-transfected cells, while PICK1 co-transfected cells showed further increase in cell death. ***B***, The percentage of cell death was quantified from multiple experiments. The extent of cell death after acid treatment is expressed as a percentage of that from pH7.4 solution incubation. ASIC1a transfection significantly increased cell death compared with non-transfected cells or PICK1-only transfected cells. Co-transfection with PICK1 further enhanced ASIC1a-mediated cell toxicity (* p < 0.05, ** p < 0.01, *** p < 0.001).

To gain insight into the functional significance of PICK1-regulated trafficking of ASIC1a, we examined PICK1's role in acidosis-induced cell toxicity. We transfected ASIC1a, with or without PICK1, into COS7 cells. Forty eight hours after transfection, cells were treated with pH7.4 or pH6.0 solution for 2, 4 or 6 hours. We then quantified the percentage of cells that show condensed nuclei, a marker for apoptotic cell death. Similar to earlier reports [11,44,45], we found that ASIC1a overexpression significantly increased cell death upon acid treatment (ASIC1a transfected cells: 145.8% ± 14.8%, 164.6% ± 8.9%, 187.0% ± 12.8% for 2, 4, 6 hours respectively. n = 7, * p < 0.05, ** p < 0.01 compared to control groups, Figure 4). Co-expression of PICK1 with ASIC1a further increased acidosis-induced cell death (ASIC1a + PICK1 transfected cells: 155.0% ± 11.4%, 192.0% ± 9.0%, 244.0% ± 16.6% for 2, 4, 6 hours respectively. n = 10, ** p < 0.01, *** p < 0.001 compared to control groups; * p < 0.05 when compared with ASIC1a-transfected cells, Figure 4). In contrast, in non-transfected cells or PICK1-only transfected cells, pH6 solution treatment did not significantly affect cell viability (non-transfected cells: 104.5% ± 10.0%, 100.8% ± 15.8%, 99.0% ± 12.3% for 2, 4, 6 hours respectively; PICK1-transfected cells: 90.2% ± 14.0%, 104.4% ± 16.5%, 103.0% ± 17.1% for 2, 4, 6 hours respectively. n = 5, Figure 4). These results indicate that PICK1 does not increase acidotoxicity by itself. Instead, it increases the acidotoxicity mediated by ASIC1a by increasing the level of ASIC1a at cell surface.

**Figure 5:**
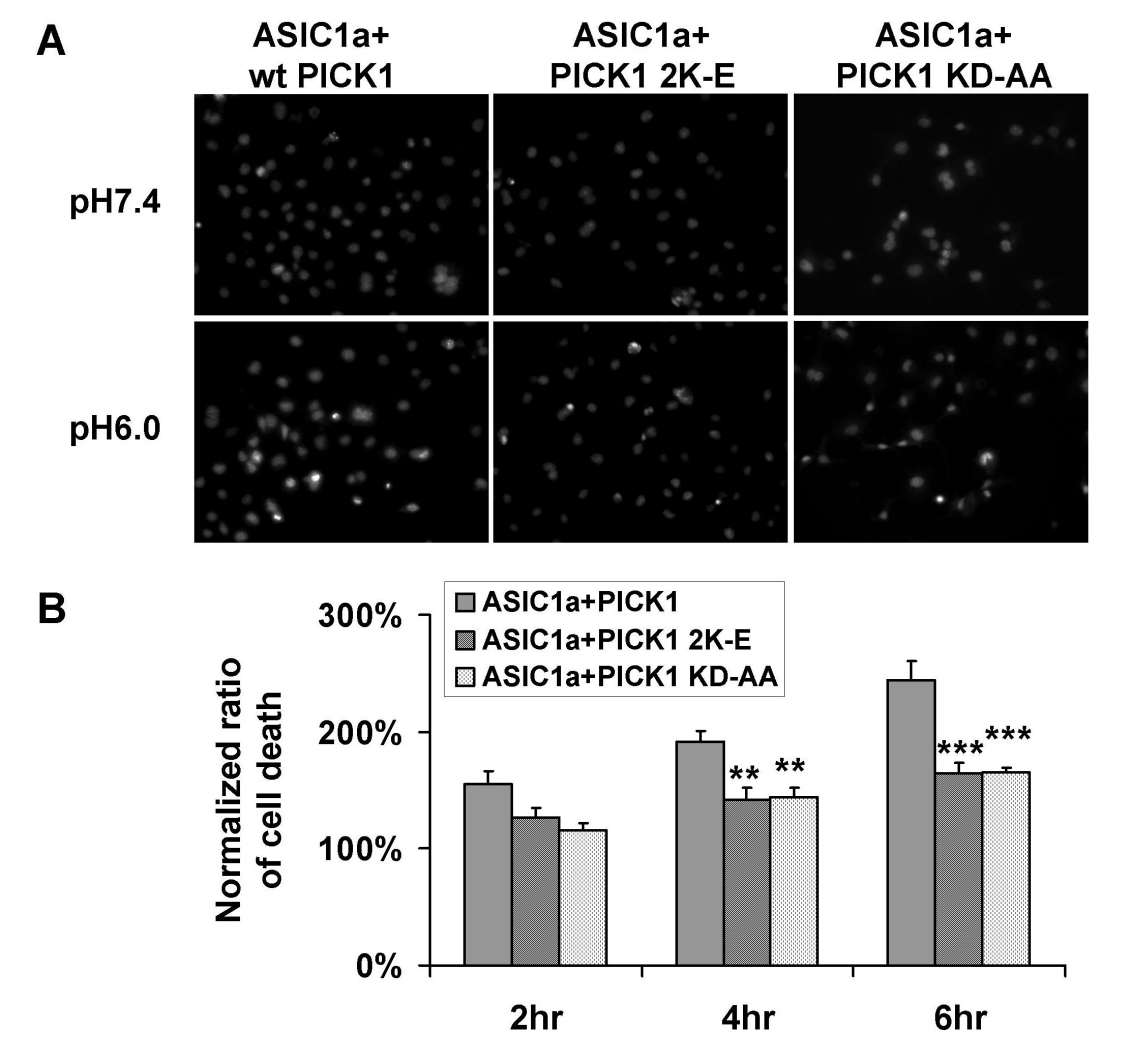
**Lipid binding of PICK1 is required for regulating ASIC1a-mediated acidotoxicity**. ***A***, ASIC1a with wild-type PICK1, lipid binding-deficient mutant PICK1 2K-E or PDZ mutant PICK1 KD-AA were transfected into COS7 cells. Cell toxicity study was then performed as in Fig. 5. ***B***, The percentage of cell death was quantified from multiple experiments. Wild-type PICK1 co-transfection showed significantly more cell death in response to acidosis compared with mutant PICK1 co-transfection (** p < 0.01, *** p < 0.001). There was no further increase of cell death in PICK1 2K-E and PICK1 KD-AA co-transfected cells compared with ASIC1a-only transfected cells (p > 0.05).

Both the PDZ and BAR domains are required for PICK1's effect on surface expression of ASIC1a, we therefore asked if the PDZ and BAR domain mutants abolish the potentiation effect of PICK1 on acid-induced cell death. As expected, when co-expressed with ASIC1a, both PICK1 2K-E (ASIC1a + PICK1 2K-E: 126.9% ± 7.8%, 141.5% ± 10.7%, 164.0% ± 9.6% for 2, 4, 6 hours respectively. N = 6, ** p < 0.01, *** p < 0.001 compared to wild-type PICK1 and ASIC1a co-transfected groups, Figure 5) and PICK1 KD-AA had significantly lower death rate comparing to wild-type PICK1 (ASIC1a + PICK1 KD-AA: 115.9% ± 5.8%, 144.3% ± 8.0%, 164.7% ± 4.7% for 2, 4, 6 hours respectively, n = 6, ** p < 0.01, *** p < 0.001 compared to wild-type PICK1 and ASIC1a co-transfected groups, Figure 5). These data indicate that PICK1 enhanced ASIC1a-mediated cell toxicity and this enhancement is dependent on PICK1's lipid binding ability and its interaction with ASIC1a.

## Discussion

ASIC1a-mediated acidotoxicity has emerged as an important concept for understanding the mechanism of brain injury in multiple disease paradigms [46,47]. Our result that PICK1 increases surface levels of ASIC1a and ASIC1a-mediated acidotoxicity provides an interesting way to regulate ASIC1a in acid-induced cell toxicity. The findings here may lead to potential means to alter surface levels of ASIC1a and subsequently, acid-induced cell toxicity.

PICK1 interacts with ASICs and this interaction was proposed to regulate the function of ASICs [38-41]. However, little is known about the mechanism of the regulation. Our results here provide the first evidence that two domains (the BAR and PDZ domain) within PICK1 cooperatively regulate ASIC1a, and consequently its surface expression. Consistent with earlier results [38,39], a direct interaction between PICK1 and ASIC1a through the PDZ interaction is important. In addition, and similar to our earlier studies on AMPA receptors [36], the effect of PICK1 on ASIC1a localization and surface also requires the lipid binding of PICK1. When the BAR domain of PICK1 was mutated, it can no longer induce ASIC1a clustering or change its surface levels, although the interaction between the two proteins was unaffected.

These findings provide a mechanistic understanding of PICK1's role on ASIC1a trafficking and acidosis-induced cell death. It is possible to change surface levels of ASIC1a and thus ASIC1a-mediated acidotoxicity, by perturbing either the protein-protein interaction or protein-lipid interaction, for instance, via protein kinase or lipid kinase pathways. In fact, protein kinase A was reported to phosphorylate ASIC1a and inhibit its interaction with PICK1 [41], while protein kinase C was reported to enhance ASIC2a-mediated current via PICK1 [40]. Of note, the major lipid molecules that bind to PICK1 are phosphoinositides, which are dynamically modified by lipid kinase and phosphatase on the cell membrane [36]. It would be interesting to test whether these protein kinases/phosphatases or lipid kinases/phosphatases play any role in ASIC1a trafficking and/or acidotoxicity.

In addition to PICK1, a number of other PDZ domain-containing proteins have been found to interact with ASICs. A multivalent PDZ domain containing-protein CIPP (channel-interacting PDZ domain protein) was found to interact with ASIC3 and overexpression of CIPP potentials ASIC3-mediated currents [48]. This potentiation is also likely due to increased surface expression of ASIC3. PSD-95 and several other PDZ domain-containing proteins including Lin-7b, MAGI-1b and PIST were also found to interact with ASIC2a and ASIC3 [6,39]. While PSD-95 decreased surface ASIC3 and ASIC3-mediated currents, Lin-7b increased surface ASIC3 and ASIC3-mediated current. These results, together with ours, suggest that it could be a general mechanism for PDZ domain-containing proteins to regulate surface expression and subcellular targeting of ASICs. Interestingly, while previous studies show that ASIC1a is present in dendritic spines and enriches in synaptosomal preparations [5,6], the mechanisms for synaptic targeting of ASIC1a remain unclear. In two reports, ASIC1a did not coIP with PSD-95 [6,49]. Our results here may provide one possible mechanism for regulating ASIC1a targeting to synapses. It will be interesting to address the question if manipulating PICK1 changes ASIC1a synaptic localization in the future.

Previous studies have shown that PICK1 regulates multiple neuronal receptors/transporters. Similar to what is reported here, PICK1 increases surface dopamine transporters (DATs) and enhances DAT uptake [50]. In contrast, overexpression of PICK1 decreases surface expression of AMPA receptors [31,33,36,51] and netrin-1 receptor UNC5H1 [52]. The detailed mechanism of PICK1-mediated protein trafficking is not clear at this moment. One model which could reconcile these differences is that PICK1 may maintain an intracellular reserve pool of membrane proteins. This pool of membrane proteins will engage in exchanging with cell surface proteins in a regulated manner. Consistent with this speculation, knockout of PICK1 leads to deficiency in both the insertion of GluR2 to cell surface of cerebellar stellate cells [28] and the removal of GluR2 from cell surface of cerebellar Purkinje neurons [27]. Upon stimulation, PICK1 can facilitate either endocytosis or exocytosis of its binding partners, depending on the distribution of these proteins in different pools and the nature of the stimulation. While the detailed mechanisms need more clarification, these findings suggest that PICK1 is a common protein trafficking regulator that couples membrane proteins to trafficking machinery via its unique combination of the PDZ domain and BAR domain.

## Methods

### cDNA cloning, mutagenesis and protein purification

Rat PICK1 cDNAs [26] and human ASIC1a (long form [38], kindly provided by Dr. Garcia-Anoveros and Dr. Corey) constructs were subcloned into corresponding expression vectors in frame by restriction enzyme digestion and ligation. Mouse ASIC1a expression constructs have been described earlier [7,24]. To generate BAR domain mutants of PICK1, we synthesized PCR (polymerase chain reaction) primers containing the desired mutation. PCR mutagenesis was performed using a Quikchange site-directed mutagenesis kit (Stratagene, La Jolla, CA, USA). All constructs were subsequently confirmed by sequencing. To produce fusion proteins, cDNA constructs were transformed into *Escherichia Coli *BL21 cells and induced with IPTG (isopropyl-beta-D-thiogalactopyranoside). GST (glutathione S transferase) fusion proteins were affinity-purified by glutathione-Sepharose-4B (Amersham Biosciences, Uppsala, Sweden) and His fusion protein was affinity-purified by Ni^2+ ^chelate resin nickel-nitrilotriacetic acid (Qiagen, Valencia, CA, USA), according to the manufacturers' instructions. Purified fusion proteins were eluted and dialyzed against the corresponding buffers for follow-up experiments. Fusion protein concentrations were determined by Coomassie assays (Pierce, Rockford, IL, USA).

### ASIC1 antibody

An anti-ASIC1 antibody was generated by injecting rabbits with a bacterially expressed GST fusion protein containing the amino acids 513-574 of human ASIC1a. Antiserum was purified by passing through Affi-Gel 10 (Bio-Rad, Richmond, CA, USA) that was coupled to His tagged C-terminal ASIC1a fusion protein (containing amino acids 513-574), washing with Tris-buffered saline (TBS, pH7.4), eluting with 100 mM glycine-HCl pH 2.8, and neutralizing with TBS (pH 8.0). A rabbit anti-ASIC1 antibody was kindly provided by Dr. John Wemmie and has been described earlier [10].

### Cell culture, transfection and immunostaining

Human embryonic kidney (HEK) 293T cells or monkey kidney cells (COS7) were cultured in MEM (modified Eagle's Medium) media (Invitrogen-Gibco, Grand Island, NY, USA) plus fetal bovine serum. For immunostaining, HEK293T cells were grown on coverslips coated with 0.2% gelatin. cDNA constructs were transfected into the HEK293T cells by calcium phosphate co-precipitation. The cells were fixed 36-48 hours after transfection by 4% paraformaldehyde and 4% sucrose in phosphate-buffered saline (PBS) for 20 minutes at room temperature. The cells were then permeabilized by 0.2% Triton X-100 in PBS for 10 minutes at room temperature. After blocking with 10% normal donkey serum (NDS) in PBS for 1 hour, the cells were incubated with affinity-purified rabbit anti-ASIC1 antibody in 3% NDS for 1 hour at room temperature, followed by 1 hour of incubation with Red-X conjugated fluorescence anti-rabbit secondary antibody (Jackson Immunoresearch; West Grove, PA, USA). After washing with PBS, the coverslips were mounted with Permafluor (Immunon, Pittsburgh, PA, USA). The cells were observed with a Nikon Eclipse TE2000 (Nikon Co., Tokyo, Japan) inverted fluorescence microscope under a 60x Plan Apochromatic oil lens (NA = 1.4, Nikon Co.). Pictures were taken by a monochrome low noise cooled CCD camera (SPOT-RT, Diagnostic Instruments, Sterling Heights, MI, USA) controlled by Metamorph imaging acquisition software (Universal Imaging, West Chester, PA, USA). Images were processed with Adobe Photoshop to adjust intensity and contrast, to select the region of interest and to overlay two images. All images were taken in monochrome gray scale and artificially colored for presentation.

### Co-immunoprecipitation

GFP-tagged human ASIC1a were co-transfected into HEK293T cells with myc-PICK1, myc-PICK1 2K-E or myc-PICK1 KD-AA. Two days after transfection, the 293T cells were lysed with 2% Triton X-100 in PBS and incubated with anti-myc antibody/Protein A complex at 4°C for at least 2 hours. The resin was washed once with cold PBS and 1% Triton X-100, twice with cold PBS, 1% Triton X-100 and 500 mM NaCl and three times with cold PBS. After washing, the resin was eluted with 1× SDS (sodium dodecyl sulphate) sample buffer and was analyzed by SDS-PAGE (SDS polyacrylamide gel electrophoresis) and immuno-blotted with affinity-purified rabbit anti-GFP or PICK1 antibody.

### Biotinylation assay

HEK293T cells were washed three times with phosphate-buffered saline supplemented with 0.5 mM CaCl_2 _and 0.5 mM MgCl_2 _(B buffer) and treated with 0.5 mg/ml sulfo-succinimidyl-6-(biotinamido) hexanoate (sulfo-NHS-LC-biotin from Pierce) in B buffer for 5 minutes at room temperature. The free sulfo-NHS-LC-biotin was removed by rapidly washing the cells two times with 100 mM glycine in B buffer followed by two washes with B buffer. The biotinylated cells were solubilized with 1 ml RIPA buffer (10 mM Tris, pH 7.4, 150 mM NaCl, 1 mM EDTA, 0.1% SDS, 1% Triton X-100, 1% sodium deoxycholate). The samples were centrifuged at maxi-speed in a table-centrifuge for 15 minutes at 4°C. A sample of this supernatant was removed for estimation of the total protein. The remaining supernatant proteins were incubated with 100 μl 50% slurry of NeutrAvidin beads (Pierce) for 1 hour at 4°C with constant rotation. After several washes, the biotinylated surface proteins were eluted from the NeutrAvidin beads in 100 μl of 1× SDS sample buffer. The samples were subjected to SDS-PAGE and Western blot analysis.

### Hoechst staining

ASIC1a and myc-PICK1 were transfected into COS7 cells using lipofectamine 2000 (Invitrogen). Forty eight hours after transfection, the cells were incubated in a HEPES buffer (10 mM HEPES, 10 mM glucose, 140 mM NaCl, 5 mM KCl, 2 mM CaCl_2_, 0.8 mM MgCl_2_) with pH7.4 or pH6.0 for different times. Chromatin condensation was detected by nucleus staining with Hoechst 33342. Briefly, cultured cells were washed once with PBS and fixed with 4% paraformaldehyde plus 4% sucrose in PBS for 15 minutes at room temperature. The cells were then stained with Hoechst 33342 (5 μg/ml) for 15 minutes at room temperature and washed twice with PBS. The nuclei were visualized under a fluorescence microscope at 20× magnification. The percentages of cell death were first calculated for each group and then normalized to the group incubated with pH7.4 solution. The extent of cell death after acid treatment was expressed as a percentage of the group that incubated with pH7.4 solution at the same time.

### PNGase and EndoH treatment and western blot analysis

Culture and lipofectamine 2000-mediated transfection of Chinese hamster oocyte (CHO) cells (purchased from ATCC) were done as described earlier [24]. Mouse ASIC1a was co-transfected with eGFP or GFP-PICK1. Two days after transfection, cells were scraped off in PBS containing 1%NP40 and 0.5%SDS in the presence of protease inhibitors (Roche). Cell lysates were homogenized by brief sonication and cleared by centrifugation. For deglycosylation, cell lysates were boiled for 10 min at 100°C in 1× glycoprotein denaturing buffer (supplied by the manufacture), followed by adding 1.5 μl of PNGase F (= 11.5 mU, New England Biolabs) or 1 μl of Endo H (= 50 mU, New England Biolabs) to 50 μl of lysate, and incubated overnight at 37°C. Untreated and treated lysates were analyzed by western blot, using an Odyssey imaging system (LiCor) as described earlier [24].

### Statistics

Student *t *test was used for statistics unless otherwise specified.

## List of abbreviations

ASIC: acid-sensing ionic channel; AMPA: alpha-amino-3-hydroxy-5-methyl-4-isoxazole propionate; BAR domain, Bin/Amphiphysin/Rvs domain; CaMKII: Ca^2+^/calmodulin-dependent protein kinase II; CIPP: channel-interacting PDZ domain-containing protein; DAT: dopamine transporter; DMSO: dimethyl sulfoxide; ENaC/DEG: epithelial Na^+ ^channel/degenerin; GST: glutathione S transferase; HEK293 cells: human embryonic kidney 293 cells; IPTG: isopropyl-beta-D-thiogalactopyranoside; MEM: modified Eagle's medium; NDS: normal donkey serum; NMDAR: N-methyl-D-aspartate receptor; PBS: phosphate buffered saline; PCR: polymerase chain reaction; PICK1: protein interacting with C kinase 1; SDS: sodium dodecyl sulphate; SDS-PAGE: SDS polyacrylamide gel electrophoresis; TBS: Tris-buffered saline; TPA: tissue plasminogen activator.

## Competing interests

The authors declare that they have no competing interests.

## Authors' contributions

WJ, CS and LJ performed the experiments and analyzed data, XMZ and JX participated in the design, data analysis and coordination of the study, WJ, CS, XMZ and JX wrote the manuscript. All authors read and approved the final manuscript.
